# The longitudinal effect of ejaculation on seminal vesicle fluid volume and whole-prostate ADC as measured on prostate MRI

**DOI:** 10.1007/s00330-017-4905-x

**Published:** 2017-07-04

**Authors:** Tristan Barrett, James Tanner, Andrew B. Gill, Rhys A. Slough, James Wason, Ferdia A. Gallagher

**Affiliations:** 1 Department of Radiology, Addenbrooke’s Hospital and University of Cambridge, Cambridge, CB2 0QQ UK; 2 CamPARI Clinic, Addenbrooke’s Hospital and University of Cambridge, Cambridge, CB2 0QQ UK; 3 MRC Biostatistics Unit, University of Cambridge, Cambridge, CB2 0QQ UK

**Keywords:** Prostate, MRI, Seminal vesicles, Preparation, Ejaculation

## Abstract

**Objective:**

To prospectively investigate the longitudinal effect of ejaculatory abstinence on MRI-measured seminal vesicle (SV) volume and whole-prostate ADC over consecutive days.

**Methods:**

15 healthy male volunteers (mean 35.9 years, range 27–53) underwent 3-T MRI at baseline and 1, 2 and 3 days post-ejaculation. Prostate and SV volumes were derived by volume segmentation and whole-gland apparent diffusion coefficient (ADC) values calculated. A mixed-effects linear regression compared ADC values and prostate/seminal vesicle volumes in each volunteer between studies in a pairwise manner.

**Results:**

All subjects completed the four MRIs. Mean prostate volume was 22.45 cm^3^ (range 13.04–31.21 cm^3^), with no change between the four studies (*p* = 0.89–0.99). 13/15 subjects showed SV volume reduction from baseline to day 1, with group-mean decreasing from 6.45 to 4.80 cm^3^ (−25.6%, *p* < 0.001), and a significant reduction from baseline to day 2 (−18.1%, *p* = 0.002). There was a significant volume increase from both day 1 (+21.3%, *p* = 0.006) and day 2 (+10.2%, *p* = 0.022) to day 3 post-ejaculation. There was a significant reduction in ADC from 1.105 at baseline to 1.056 × 10^−3^ mm^2^/s at day 1 (mean −4.3%, *p* = 0.009).

**Conclusion:**

The longitudinal effect of ejaculation on SV volume was demonstrated. Significant reductions in SV volume and whole-gland ADC were observed post-ejaculation, supporting a 3-day period of abstinence before prostate MRI.

***Key Points*:**

• *Seminal vesicle volume significantly reduced 24 h post-ejaculation remaining reduced at day 2*

• *Seminal vesicle fluid volume significantly increased from day 1 to day 3 post-ejaculation*

• *There was a significant reduction in whole-gland prostate ADC values day 1 post-ejaculation*

• *3-day abstinence from ejaculation is required to ensure maximal seminal vesicle distension*

**Electronic supplementary material:**

The online version of this article (doi:10.1007/s00330-017-4905-x) contains supplementary material, which is available to authorized users.

## Introduction

Multiparametric MRI is increasingly being used for detection, localisation and staging of prostate cancer [1]. Determining whether tumour extends into the seminal vesicles (T3b disease) is a key component for prostate cancer staging, with implications for risk stratification, management and longer-term prognosis. Curative treatment is more likely if the disease is organ-confined, with no evidence of extracapsular extension, seminal vesicle invasion or distant disease, and optimal assessment of T3b disease is thought to require maximal distension of the seminal vesicles [2]. Seminal vesicle (SV) distension may also be important for radiation therapy planning: target volumes for radical radiotherapy treatment incorporate the proximal 2 cm of SV in low- to intermediate-risk patients or the entire seminal vesicles in patients with high-risk disease or suspected T3b involvement [3]. The target volume will therefore be defined by the degree of SV distension and may subsequently change between radiotherapy sessions if prior ejaculatory status has an effect.

In order to maintain maximum distention of the seminal vesicles, some centres recommend that patients refrain from ejaculation for 3 days prior to the MRI study to achieve maximum distension [4, 5]. However, there is no consensus recommendation, with preparation times varying between centres [6, 7]. The recently updated Prostate Imaging Reporting and Data System (PI-RADS) guidelines note that some centres recommend refraining from ejaculation for 3 days prior to the MRI, but highlight a lack of objective evidence for such areas of patient preparation [8].

Only one prospective MRI study has documented volume change in this context, demonstrating a 41% mean reduction in SV volume immediately post-ejaculation [9]. Scanning at this time point is not representative of a clinical MRI study and it is in fact possible that seminal vesicle fluid replacement may occur relatively quickly. This study also showed a significant 14% reduction in peripheral zone apparent diffusion coefficient (ADC) values, which may be important for lesion detection and therefore warrants further investigation. Another retrospective study compared two discrete groups of patients where the last ejaculation was either more than or less than 3 days prior to the MRI [10]. SV volume was measured by planimetry and was found to be significantly higher in those refraining from ejaculation for greater than 3 days. The two groups were age-matched; however, this may not fully account for the known considerable interpatient variability in seminal vesicle volume [11]. Additionally, the time between ejaculation and imaging in the longer abstinence group was uncontrolled. There is currently no published longitudinal MRI data on the volume of seminal vesicles in relation to ejaculation. The purpose of this study was therefore to prospectively investigate the longitudinal effect of ejaculatory abstinence on MRI-measured seminal vesicle volume and whole-prostate ADC over consecutive days, in normal volunteers.

## Methods

Fifteen healthy male volunteers (mean age 35.9 years, median 34, range 27–53) were included in this prospective, institutional review board-approved study (Reference: CUH 15/YH/0570). Participants were recruited through posters on campus between March and June 2016, with written informed consent obtained in all cases. MR imaging was performed on four consecutive days. The subjects were instructed to abstain from ejaculation for at least 3 days prior to the first MRI, to perform ejaculation after scan 1 and prior to scan 2, then refrain from ejaculation until the completion of the study. Scans 2, 3 and 4 were therefore performed 1–24 h, approximately 48 h and 72 h post-ejaculation, respectively.

### Magnetic resonance imaging

MR imaging was performed on a 3-T MR750 magnet (General Electric Healthcare, Waukesha, USA) using a 32-channel phased array body coil. Sequences included high resolution axial T2-weighted fast recovery fast spin echo (FRFSE) imaging, TR/TE of 3663/102 ms field of view (FOV) 22 × 22 cm, 3 mm slice thickness with no gap, in-plane resolution 0.85 × 0.57 mm, and 3 signal averages; sagittal T2 cube sequence, 1 mm slice thickness with no gap, in-plane resolution 1.0 × 0.8 mm. Axial diffusion-weighted imaging (DWI) was matched to the T2 axial sequence, using a dual spin-echo planar pulse sequence with TR/TE of 3775/70 ms, FOV 28 × 28 cm, resolution 2.2 × 2.2 mm, 6 signal averages and *b* values of 150, 750, 1000 and 1400 from which automated ADC maps were generated (Table 1).

**Table 1:** Sequences in MRI protocol

Parameter	Axial T2 2D FSE	Sagittal T2 3D FSE	EPI-2D DWI
TR/TE (ms)	3663/102	3000/117	3775/70
Flip angle (°)	111	160	90
ETL length	16	90	1
Averages	3	2	6
*b* value	N/A	N/A	150, 750, 1000, 1400
Section thickness (mm)	3	1	3
Section gap (mm)	0	0	0
FOV (mm)	220	220	280
Resolution (mm)	0.85 × 0.57	1.0 × 0.8	2.2 × 2.2
Acquisition time (min)	6:07	3:13	02:42

*FSE* fast spin echo, *EPI* echo planar imaging, *DWI* diffusion-weighted imaging, *FOV* field of view, *ETL* echo train length

### MR segmentation

Seminal vesicle (SV) and prostate volumes were calculated using whole volume segmentation on T2-weighted images using in-house software programmed in Matlab (Supplemental data 1). Whole-gland ADC measurements were acquired after outlining the prostate using the relevant T2-weighted axial sequence as an anatomical reference. ADC values were recorded from each voxel with the regions of interest (ROIs) and, after summation of values from all slices, a whole-gland ADC mean value was derived. Whole-gland T2 signal intensity was recorded from prostate outlines normalised to muscle signal intensity as an internal reference. Three ROIs were drawn within the left obturator internus muscle (≥0.5 cm^3^) on consecutive slices, with a ratio of median whole-prostate to muscle T2 signal intensity recorded for each study. All outlining was performed by a single uro-radiologist (T.B.) with 7 years’ experience reporting prostate MRI. Outlines were drawn in a random order and blinded to the clinical information of study number and time pre- or post-ejaculation.

**Fig. 1:**
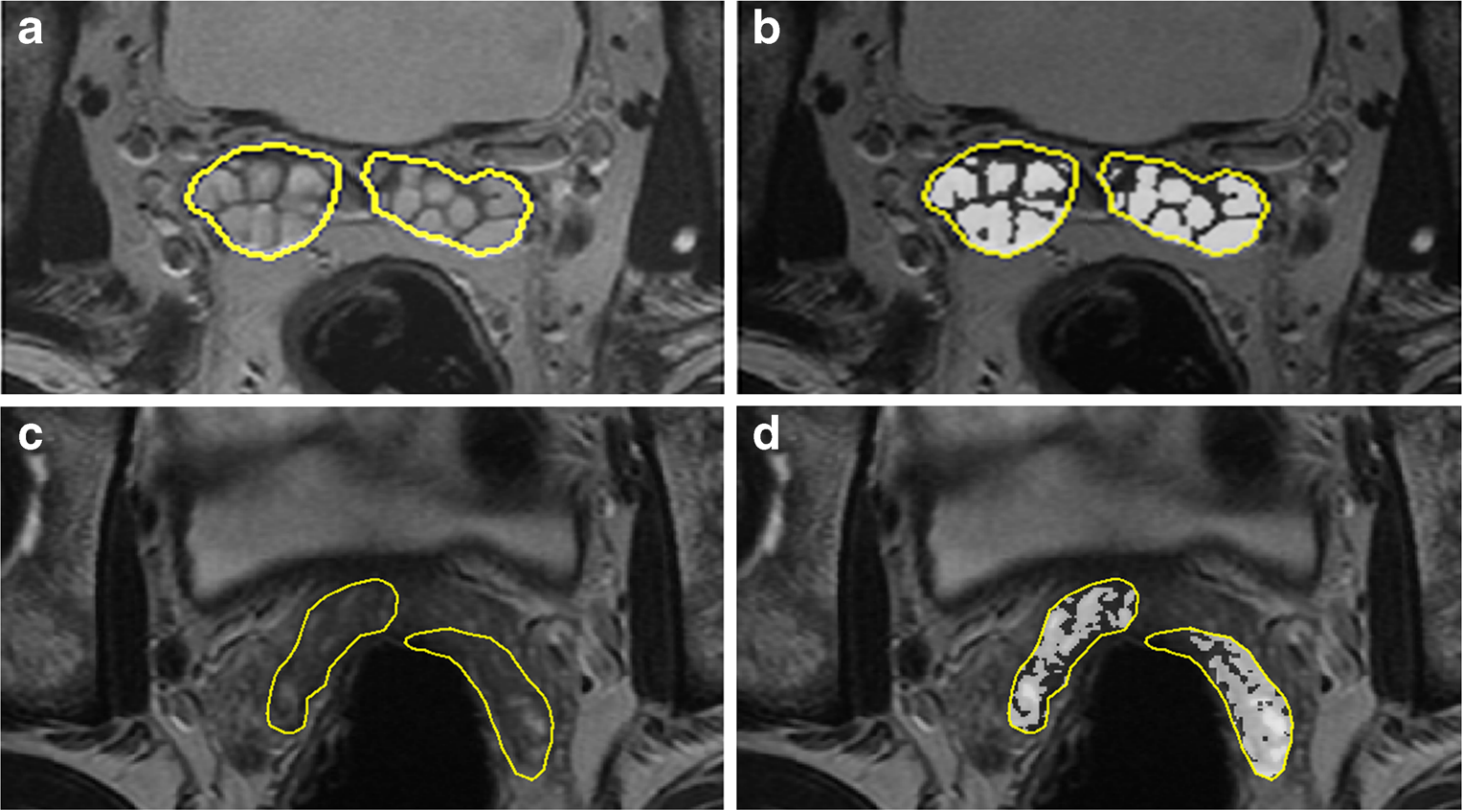
Calculating seminal vesicle fluid volume. Manual regions of interest (*yellow*) were drawn outlining the seminal vesicles on all slices for segmentation (**a**, **c**). To remove the SV wall component, the maximum signal in the central outlined area was identified and a threshold applied for inclusion of pixels > (*S*_max_ × *f*). The fraction (*f*) was set at either 0.8 (**b**) or 0.7 (**d**) for cases where motion blurring was present

Organ volumes (in cubic millimetres) were calculated from a sum of the drawn ROIs (in square millimetres) multiplied by the spacing between acquired slices (in millimetres). An alternative method was also employed incorporating quadratic interpolation in the form of Simpson’s rule applied in the slice direction when summing ROI areas on adjacent slices. The two methods were compared for approximate equality of results as a quality control check. The subsequent analysis was then performed on the interpolated volume measurements. For SV volume, a separate calculation was made of fluid volume by excluding the wall volume which is expected to remain constant, but contributes proportionately more to the overall volume when the seminal vesicles are collapsed or underfilled. As the seminal vesicles contain fluid and organ wall in a convoluted pattern which is difficult to outline manually, a thresholding method was applied to yield a non-connected subset of areas which excluded the wall volume. The threshold on image intensity was set to a fraction (*f*) of the maximum pixel intensity (*S*_max_) calculated as the 95% percentile in the SV ROI intensity histogram. Thus, for any given organ, the maximum signal in the central outlined slice was found and the threshold for inclusion of pixels set to S > (*S*_max_ × *f*). The fraction *f* was varied manually at limits of 0.7 or 0.8 (depending on the presence of motion-induced ‘blurring’) to achieve optimal segmentation as evaluated through visual inspection by a radiologist (T.B.) blinded to the clinical information (Fig. 1). Fluid volume was then calculated in the same way as described above, multiplying the thresholded total of ROI subareas by the spacing between slices.

### Statistics

A linear mixed-effects model was fitted to the seminal vesicle fluid volume and whole-prostate ADC observations. Each model included fixed effects for the time of the observation (baseline, day 1, day 2 or day 3) and random effects for each individual to account for correlation within individuals. A log transform was applied to the data to ensure homoscedastic error variance. A paired two-tailed *t* test was used to compare the ratio of T2 signal intensity to muscle between studies in a stepwise manner. Spearman’s correlation was performed to assess the relationship between age and both prostate and seminal vesicle volume and between time post-ejaculation and change in SV volume from baseline to day 1. Statistical analyses were performed using Stata®14 (StataCorp LP, Texas, USA). *p* values of less than 0.05 were considered to be statistically significant.

## Results

All 15 volunteers completed each of the four scans at the appropriate time. Study 2 (within 24 h of ejaculation) was performed at a mean of 11.13 h post reported ejaculation (median 11, range 2–21, interquartile range 8–15 h). For one subject, there was a technical failure of diffusion-weighted imaging on the day 1 scan and the results were excluded from the ADC analysis.

### Volume measurements

The mean prostate volume measured on day 3 was 22.45 cm^3^ (median 23.28 cm^3^, range 13.04–31.21 cm^3^). No correlation was demonstrated between prostate volume and age; Spearman’s rank Rho −0.035. There was no change in whole-gland prostate volume between the four studies: baseline, 22.46 cm^3^; day 1, 22.47 cm^3^; day 2, 22.53 cm^3^ and day 3, 22.45 cm^3^ (*p* = 0.89–0.99); Table 2.

**Table 2:** Mean volume changes over time

Anatomical Region	Baseline	Day 1	Day 2	Day 3
Seminal vesicle total	12.77 (5.53)	11.41 (5.81)	11.89 (6.48)	12.47 (5.98)
Seminal vesicle wall	6.31 (2.38)	6.61 (2.97)	6.59 (2.61)	6.65 (3.05)
Seminal vesicle fluid	6.45 (3.48)	4.8 (3.29)	5.28 (4.2)	5.82 (3.23)
Prostate	22.46 (6.29)	22.47 (5.66)	22.53 (5.84)	22.45 (6.00)

Volumes expressed in cm^3^, standard deviation in parentheses

The average total seminal vesicle volume including wall and fluid volume was 12.47 cm^3^ (median 11.82 cm^3^, range 5.44–28.81 cm^3^) as measured on day 3, i.e. following a uniform ejaculatory preparation time. There was no significant difference in overall average volume of the right (6.35 cm^3^) and left (6.13 cm^3^) seminal vesicles, with the difference ranging from 0.21 to 2.17 cm^3^; *p* = 0.50. As expected, there was no change in SV wall volume between the studies: baseline, 6.31 cm^3^; day 1, 6.61 cm^3^; day 2, 6.59 cm^3^ and day 3, 6.65 cm^3^; *p* = 0.38–0.49.

**Fig. 2:**
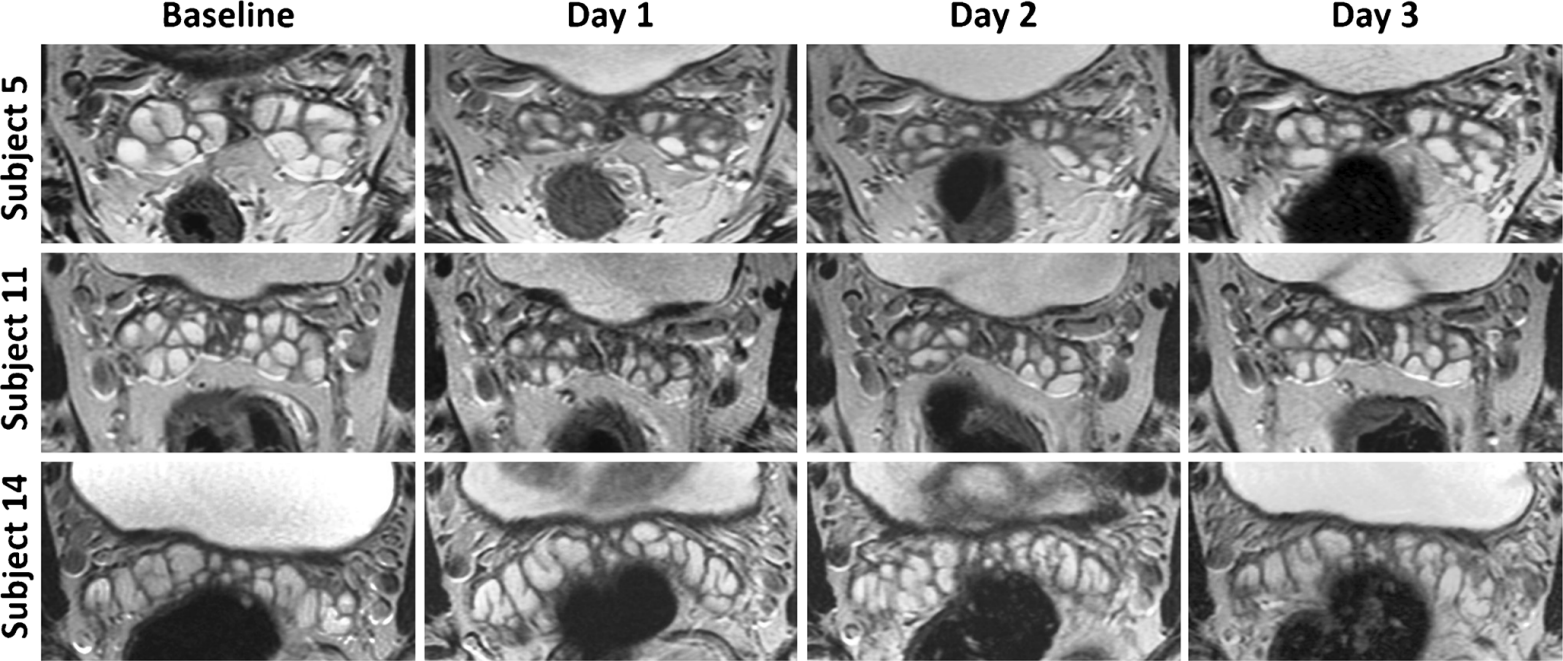
Examples of seminal vesicle volume change over time. Axial T2-weighted MR imaging performed at baseline (minimum 3 days post-ejaculation), and post ejaculation on days 1, 2 and 3. *Top row* subject 5 (aged 27) shows significant reduction in volume from a baseline of 9.7 cm^3^ to 3.6 cm^3^ on day 1 and subsequently to 4.7 cm^3^ and 6.9 cm^3^ on days 2 and 3, respectively. *Middle row* subject 11 (age 53) shows similar reductions from 7.0 cm^3^ at baseline to 3.3 cm^3^, 4.2 cm^3^ and 5.3 cm^3^ on days 2, 3 and 4, respectively. Note apparent increase in wall thickening on day 2, due to relative underdistension. *Bottom row* subject 14 (age 36) shows little difference in SV volume over the four scans: 10.6 cm^3^, 10.0 cm^3^, 10.5 cm^3^ and 10.9 cm^3^, respectively

**Fig. 3:**
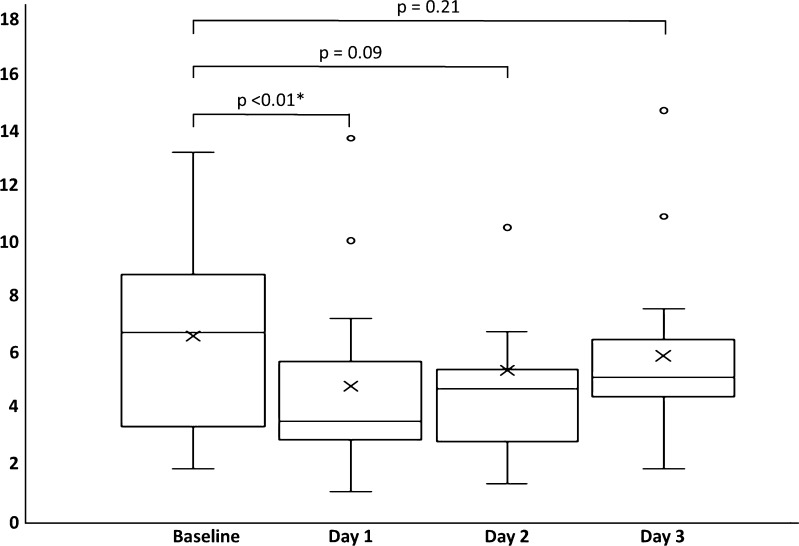
Box and whisker plots of seminal vesicle fluid volumes between each of the four scans. *Top* and *bottom* of boxes represent 25th and 75th percentiles of data, *X* in *boxes* represents mean, with *line* representing median value and *bars* representing data within 1.5 times interquartile range. *Circles* denote outliers. *p* values are shown for inter-group comparisons, *p < 0.05

The average seminal vesicle fluid volume on day 3 with a standardised preparation time was 5.82 cm^3^ (median 5.25 cm^3^, range 2.1–14.52 cm^3^). There was no significant correlation between age and seminal vesicle fluid volume, Rho 0.171. There was a reduction in seminal vesicle fluid volume in 13 of 15 subjects from baseline to day 1, with a general trend for increasing volumes on subsequent days (Fig. 2). The two patients without SV volume reduction showed volume increases of 1.05 cm^3^ (8.4%) and 0.02 cm^3^ (0.9%), respectively. There was no significant correlation between reported ejaculation time and subsequent change in SV fluid volume at day 1, Rho −0.147. SV fluid volume significantly reduced from 6.45 cm^3^ at baseline to 4.80 cm^3^ at day 1 (25.6% reduction, *p* < 0.001) and to 5.28 cm^3^ at day 2 (18.1% reduction, *p* = 0.002); Fig. 3. There was subsequently a significant increase in volume to 5.82 cm3 at day 3 post-ejaculation from 4.80 cm^3^ at day 1 (35.1% increase, *p* = 0.006) and from 5.28 cm^3^ at day 2 (10.2% increase, *p* = 0.022); Table 3.

**Table 3:** Comparison of mean seminal vesicle fluid volumes between studies

Comparison	Absolute change (cm^3^)	Percentage change (%)	Mean difference (log scale)	Confidence interval	*p* value
Baseline vs day 1	−1.65 (1.85)	−25.1 (19.9)	−0.326	(−0.494, −0.158)	<0.001*
Baseline vs day 2	−1.18 (2.5)	−19.5 (29.3)	−0.280	(−0.448, −0.112)	0.002*
Baseline vs day 3	−0.63 (1.86)	−9.77 (38.0)	−0.076	(−0.244, 0.092)	0.38
Day 1 vs day 2	0.48 (1.81)	10.0 (30.2)	0.046	(−0.122, 0.214)	0.59
Day 1 vs day 3	1.02 (1.33)	21.3 (43.3)	0.250	(0.082, 0.418)	<0.006*
Day 2 vs day 3	0.55 (1.41)	10.2 (34.0)	0.204	(0.036, 0.372)	0.022*

Standard deviation in parentheses

*p < 0.05, denoting significance

### Whole-gland T2 signal intensity measurements

The mean whole-gland T2 signal intensity normalised to muscle measured 2.45 (ratio; unitless) at baseline and 2.49 on day 1, 2.48 on day 2 and 2.46 on day 3. There was no significant difference using *t* test comparisons between any combination of days (*p* = 0.74–0.92).

### Quantitative whole-gland ADC measurements

**Fig. 4:**
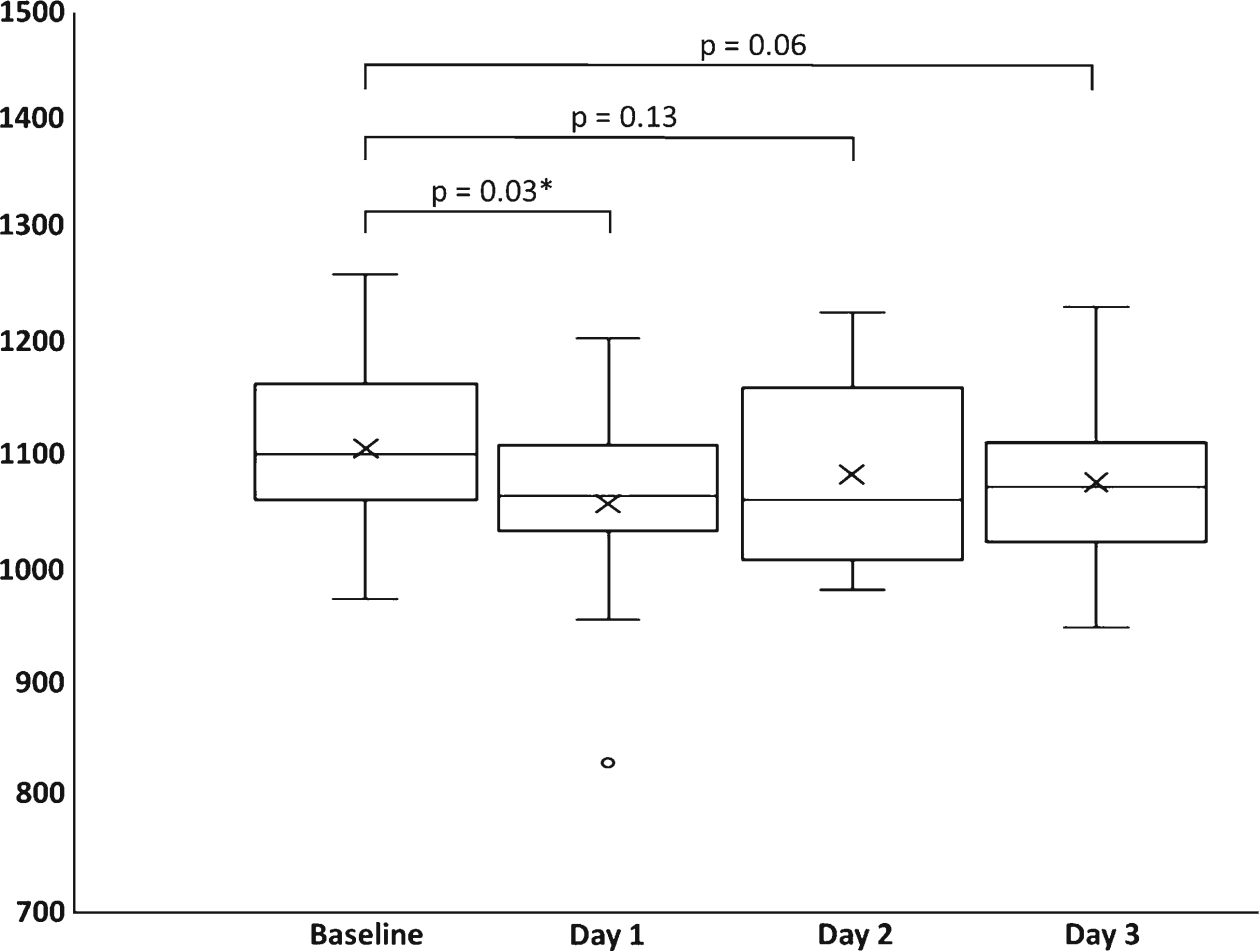
Box and whisker plots of whole-gland prostate ADC values between each of the four scans. *Top* and *bottom* of *boxes* represent 25th and 75th percentiles of data, *X* in *boxes* represents mean, with *line* representing median value and *bars* representing data within 1.5 times interquartile range. *Circles* denote outliers. *p* values are shown for inter-group comparisons, *p < 0.05

The mean whole-prostate ADC value on day 3 was 1.074 cm^3^ × 10^−3^ mm^2^/s (median 1.071, range 0.947–1.229 × 10^−3^ mm^2^/s). There was a reduction ADC value in 10 of 14 subjects from baseline to day 1 (Supplemental Table 1), with a significant mean reduction from 1.105 × 10^−3^ mm^2^/s at baseline to 1.056 × 10^−3^ mm^2^/s at day 2 (4.3% reduction, *p* = 0.009); Fig. 4. There was a general trend for ADC values to subsequently increase from day 2 to 3, but this was not significant (Table 4).

**Table 4:** Comparison of mean whole-prostate ADC values between scans

Comparison	Absolute change (10^−3^ mm^2^/s)	Percentage change (%)	Mean difference (log-scale)	Confidence interval	*p* value
Baseline vs day 1	−0.049 (0.078)	−4.29 (6.97)	−0.047	(−0.080, −0.013)	0.009*
Baseline vs day 2	−0.023 (0.054)	−2.06 (4.68)	−0.022	(−0.055, 0.011)	0.20
Baseline vs day 3	−0.031 (0.054)	−2.66 (4.77)	−0.028	(−0.061, 0.005)	0.10
Day 1 vs day 2	0.026 (0.059)	2.67 (6.44)	0.025	(−0.009, 0.058)	0.15
Day 1 vs day 3	0.019 (0.082)	2.19 (8.84)	0.018	(−0.015, 0.052)	0.28
Day 2 vs day 3	−0.007 (0.058)	−0.77 (5.69)	−0.006	(−0.039, 0.027)	0.71

Standard deviation in parentheses

*p < 0.05, denoting significance

## Discussion

This study demonstrates the longitudinal MR measurements of seminal vesicle fluid volume following ejaculatory abstinence. The overall results suggest that a period of abstinence from ejaculation is warranted prior to prostate MRI. We show a significant reduction in seminal vesicle fluid volume within 1 day of ejaculation, maintained at day 2. This is confirmed by an overall increase in volume on successive days post-ejaculation, with significantly higher volumes observed at day 3 compared to both the day 1 and 2 MRIs, thus suggesting that a minimum 3-day period of abstinence is required for maximal SV distension. A similar effect was seen for ADC values in diffusion-weighted imaging, with a significant reduction in values at day 1 post-ejaculation.

Although many centres recommend that patients refrain from ejaculation prior to prostate MR imaging, there is a lack of evidence to support this practice or to inform on the necessary period of abstinence. The rationale may be based on historical practice for investigation of male fertility, where the World Health Organisation recommends abstinence of 2–7 days for sperm analysis [12]. This is supported by work suggesting that both the semen volume (24%) and sperm density are reduced in men with abstinence of only 1 day compared to more than 4 days [13, 14]. It should be noted that although seminal vesicle fluid makes up 40–85% of the ejaculate [15], there is also a contribution from the prostate gland (15–30%), testis (<5%) and bulbourethral glands (1–5%); thus, ejaculate volume alone may not provide an accurate indication of changes in seminal vesicle volume. Early work using vesiculography [16] and ultrasound [17, 18] and subsequently confirmed by a more recent MRI study [9] showed 30–40% reductions in seminal vesicle volume immediately post-ejaculation. However, these studies are not representative of an outpatient MR patient population. A more recent study demonstrated lower seminal vesicle volumes in patients imaged less than 3 days post last ejaculation, compared to more than 3 days [10]. This study only looked at the binary cut-off of 3 days and was additionally limited by planimetry measurements rather than image-segmentation volumetry of the seminal vesicles. Furthermore, the same patients were not directly compared, and inter-patient variation, in which SV length may vary up to 11-fold [11], may therefore have affected their results.

We have demonstrated a significant reduction in whole-prostate ADC measurements within 24 h of ejaculation, and a non-significant increase in ADC between days 2 and 3. This is consistent with earlier work in the immediate post-ejaculatory phase [9]. ADC reduces with increasing Gleason grade [19, 20], which partly relates to loss of fluid in the glandular lumen [21]; higher grade tumours have also been shown to have a reduced luminal space [22]. The prostate gland contributes a small amount of fluid to the ejaculate [15] and although we did not demonstrate any appreciable change in prostate volume between studies, this fluid loss may be sufficient to have a dehydrating effect on the gland, and thus reduce the ADC value. We did not differentiate between peripheral (PZ) and transition zone (TZ) given the relative small volume of TZ and relative difficultly in zonal differentiation in this young cohort (Supplemental Fig. 1). Functional MRI imaging and in particular diffusion-weighted imaging form a key component of clinical prostate imaging and these results provide a further rationale for abstinence prior to imaging. Standardisation of patient preparation prior to prostate MRI may help to reduce inter-patient variations in ADC measurements and improve quantitative analysis, as well as addressing intra-patient variation in the context of follow-up of patients on active surveillance programs. Morgan et al. [23] showed that a greater than 10% reduction in whole-gland ADC value in patients on active surveillance predicted progression to radical treatment; if such cut-offs are to be used, this needs to be considered in light of the 5% reduction in whole-gland ADC we demonstrated at day 1. Although we did not look at the question of dynamic contrast-enhanced MRI, it is possible that this may also be affected by post-ejaculatory status, with a previous ultrasound study showing that ejaculation affected the clinical assessment of prostatitis by increasing prostatic blood flow for at least 24 h [24].

There were several limitations to our study. The study population was a small cohort of healthy volunteers who were of a younger age than typical patients with prostate cancer. It has been demonstrated that seminal vesicle volume in healthy patients decreases after the age of 60 years [25]; however, this may be partially offset by an increase in volume in patients with benign prostate hypertrophy [26]. Although these results should hold regardless of baseline volume, it is unclear whether they would be affected by an underlying disease process, or indeed whether diagnostic interpretation would be hindered. It has been suggested that the replenishment of seminal fluid in younger patients is more rapid and therefore the effects of abstinence may be less apparent [10]; while there is no direct evidence for this, it would suggest that the results demonstrated here in young volunteers would be more marked if repeated in an older age group, and further strengthens the argument for a period of abstinence prior to scanning. We did not assess dynamic contrast-enhanced MRI, which forms part of routine clinical prostate mpMRI, because the risks of repeat gadolinium deposition could not be justified in a volunteer population. Larger studies in patients with prostate cancer, ideally incorporating age-appropriate healthy controls, would serve to overcome these limitations and would also help address whether the observed variation in ADC values affects lesion conspicuity and detection.

In conclusion, we have demonstrated the longitudinal effect of ejaculation on seminal vesicle volume as measured by MR imaging. Seminal vesicle volume is significantly reduced at day 1 and 2 post-ejaculation and continues to increase at day 3. Whole-gland prostate ADC values were also found to be significantly lower post-ejaculation. The results support the rationale of a minimum 3-day period of abstinence from ejaculation prior to diagnostic prostate MRI.

## Electronic supplementary material

Below is the link to the electronic supplementary material.

ESM 1 (DOCX 1555 kb)

ESM 2 (DOCX 64 kb)

ESM 3 (DOCX 23 kb)

## References

[1] Barrett T, Turkbey B, Choyke PL (2015). PI-RADS version 2: what you need to know. Clin Radiol.

[2] de Rooij M, Hamoen EH, Witjes JA, Barentsz JO, Rovers MM (2016). Accuracy of magnetic resonance imaging for local staging of prostate cancer: a diagnostic meta-analysis. Eur Urol.

[3] Qi X, Gao XS, Asaumi J (2014). Optimal contouring of seminal vesicle for definitive radiotherapy of localized prostate cancer: comparison between EORTC prostate cancer radiotherapy guideline, RTOG0815 protocol and actual anatomy. Radiat Oncol.

[4] Bloch BN, Lenkinski RE, Rofsky NM (2008). The role of magnetic resonance imaging (MRI) in prostate cancer imaging and staging at 1.5 and 3 Tesla: the Beth Israel Deaconess Medical Center (BIDMC) approach. Cancer Biomark.

[5] Sankineni S, Osman M, Choyke PL (2014). Functional MRI in prostate cancer detection. Biomed Res Int.

[6] RadNet (2015) Multiparametric MRI. http://www.mriprostatecancer.com/prostate-mri/multiparametric-mri/. Accessed 7 Feb 2017

[7] Radboud University Nijmegen Medical Centre (2017) https://www.radboudumc.nl/en/patientenzorg/onderzoeken/mri-examination-of-the-prostate-gland. Accessed 6 Feb 2017

[8] Weinreb JC, Barentsz JO, Choyke PL (2015). PI-RADS prostate imaging-reporting and data system, 2015, version 2. Eur Urol.

[9] Medved M, Sammet S, Yousuf A, Oto A (2014). MR imaging of the prostate and adjacent anatomic structures before, during, and after ejaculation: qualitative and quantitative evaluation. Radiology.

[10] Kabakus IM, Borofsky S, Mertan FV (2016). Does abstinence from ejaculation before prostate MRI improve evaluation of the seminal vesicles?. AJR Am J Roentgenol.

[11] Gofrit ON, Zorn KC, Taxy JB, Zagaja GP, Steinberg GD, Shalhav AL (2009). The dimensions and symmetry of the seminal vesicles. J Robot Surg.

[12] World Health Organization (2010). WHO laboratory manual for the examination and processing of human semen.

[13] Marshburn PB, Giddings A, Causby S (2014). Influence of ejaculatory abstinence on seminal total antioxidant capacity and sperm membrane lipid peroxidation. Fertil Steril.

[14] De Jonge C, LaFromboise M, Bosmans E, Ombelet W, Cox A, Nijs M (2004). Influence of the abstinence period on human sperm quality. Fertil Steril.

[15] Lundquist F (1950). Aspects of the biochemistry of human semen. Acta Physiol Scand.

[16] Ichijo S, Sigg C, Nagasawa M, Siraiwa Y (1981). Vasoseminal vesiculography before and after ejaculation. Urol Int.

[17] Fuse H, Okumura A, Satomi S, Kazama T, Katayama T (1992). Evaluation of seminal vesicle characteristics by ultrasonography before and after ejaculation. Urol Int.

[18] Lotti F, Corona G, Colpi GM (2012). Seminal vesicles ultrasound features in a cohort of infertility patients. Hum Reprod.

[19] Hambrock T, Somford DM, Huisman HJ (2011). Relationship between apparent diffusion coefficients at 3.0-T MR imaging and Gleason grade in peripheral zone prostate cancer. Radiology.

[20] Donati OF, Mazaheri Y, Afaq A (2014). Prostate cancer aggressiveness: assessment with whole-lesion histogram analysis of the apparent diffusion coefficient. Radiology.

[21] Epstein JI, Allsbrook WC, Amin MB, Egevad LL, Grading Committee ISUP (2005). The 2005 International Society of Urological Pathology (ISUP) consensus conference on Gleason grading of prostatic carcinoma. Am J Surg Pathol.

[22] Lawrence EM, Warren AY, Priest AN (2016). Evaluating prostate cancer using fractional tissue composition of radical prostatectomy specimens and pre-operative diffusional kurtosis magnetic resonance imaging. PLoS One.

[23] Morgan VA, Riches SF, Thomas K (2011). Diffusion-weighted magnetic resonance imaging for monitoring prostate cancer progression in patients managed by active surveillance. Br J Radiol.

[24] Keener TS, Winter TC, Berger R (2000). Prostate vascular flow: the effect of ejaculation as revealed on transrectal power Doppler sonography. AJR Am J Roentgenol.

[25] Terasaki T, Watanabe H, Kamoi K, Naya Y (1993). Seminal vesicle parameters at 10-year intervals measured by transrectal ultrasonography. J Urol.

[26] Hayakawa T, Naya Y, Kojima M (1998). Significant changes in volume of seminal vesicles as determined by transrectal sonography in relation to age and benign prostatic hyperplasia. Tohoku J Exp Med.

