# Supplementary material for: The longitudinal effect of ejaculation on seminal vesicle fluid volume and whole-prostate ADC as measured on prostate MRI
Source: Eur Radiol. 2017 Jul 4;27(12):5236–43. doi: 10.1007/s00330-017-4905-x (PMC5674119; doi:10.1007/s00330-017-4905-x)
Supplement: Supplementary file 1 — (DOCX 1555 kb) [file 330_2017_4905_MOESM1_ESM.docx]

**
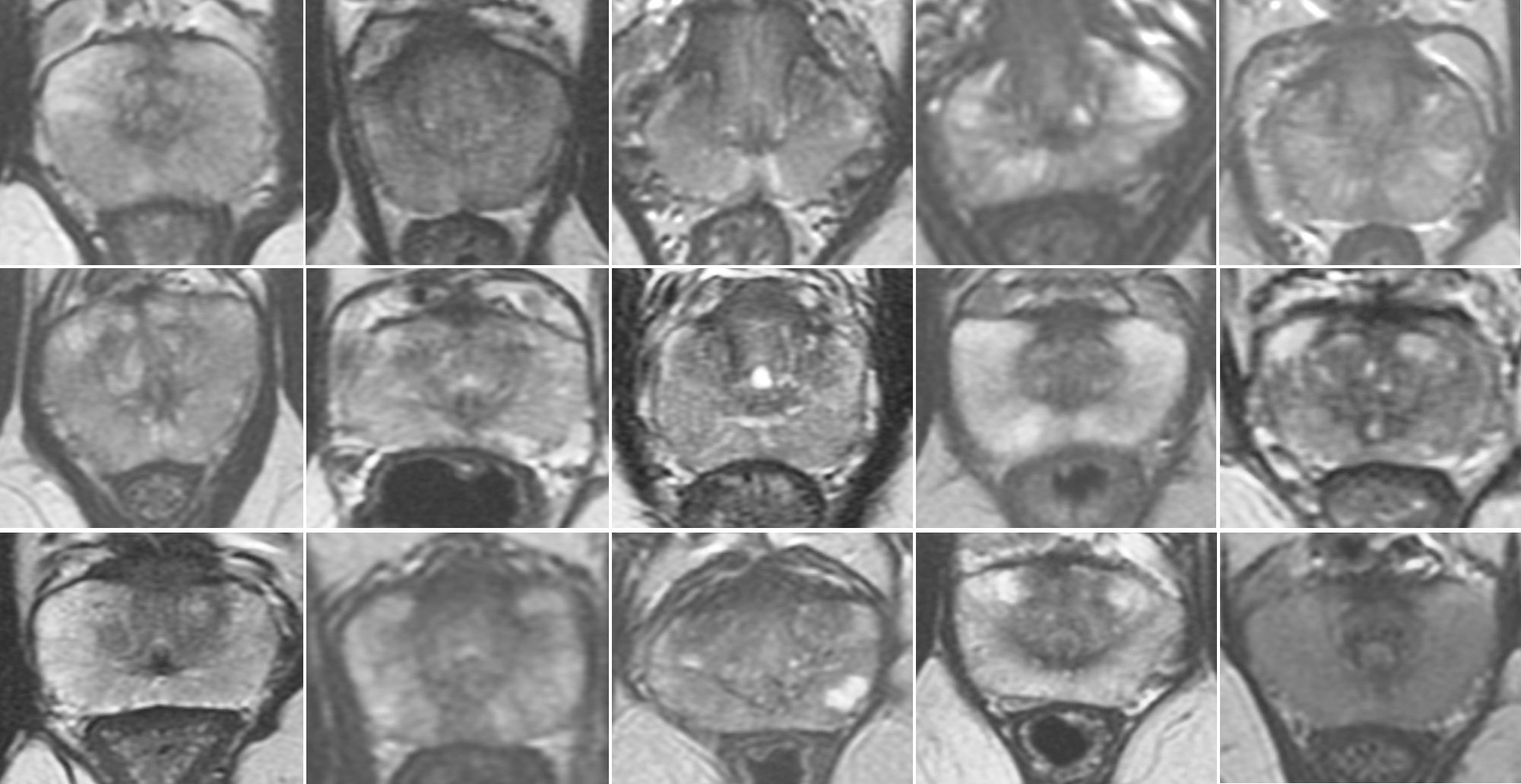
Supplemental Figure 1.** Representative T2-weighted axial images from all 15 volunteers taken at the mid gland level, highlight the minimal transition zone present and relative difficultly in assessment of zonal differentiation in some cases.
