# Supplementary material for: The longitudinal effect of ejaculation on seminal vesicle fluid volume and whole-prostate ADC as measured on prostate MRI
Source: Eur Radiol. 2017 Jul 4;27(12):5236–43. doi: 10.1007/s00330-017-4905-x (PMC5674119; doi:10.1007/s00330-017-4905-x)
Supplement: Supplementary file 2 — (DOCX 64 kb) [file 330_2017_4905_MOESM2_ESM.docx]

**Computational methods in greater detail:**

Organ volumes (mm^3^) were first calculated according to a sum of the drawn regions of interest (ROI); the area of the ROI in each slice (in mm^2^) was multiplied by the spacing between acquired slices (mm). An alternative method was also employed incorporating quadratic interpolation in the form of Simpson’s Rule. A well-known numerical method, Simpson’s Rule in effect approximates an integral (of *y* = *f*(*x*)*,* where *x*_0_, *x*_1_,… *x*_n_ are each separated by a distance *h*) by fitting adjacent sets of three *y*-values to a parabolic curve. Used here ‘*y*_i_’ is the area of the ROI on slice number ‘*i*' and ‘*h*’ is the spacing between slices. In this way the abrupt slice profile change between ROIs drawn on adjacent sets of three slices is smoothed to a parabolic function and the true organ profile and volume better approximated (see Fig. 1). In the case of the number of slices being odd (or fewer than 3), the first two *y*-values (ROI areas) were summed to a volume using the Trapezium Rule and the remainder (if any) using Simpson’s Rule.


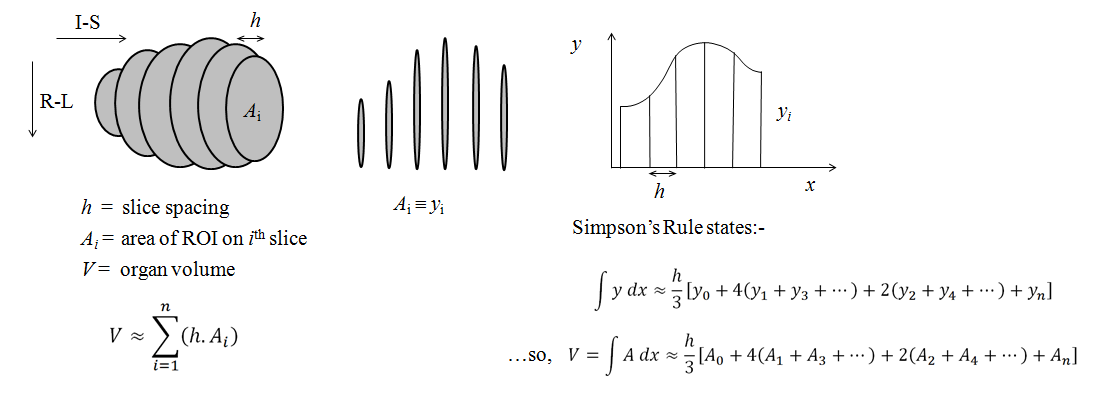


**Fig 1.** Simpson’s Rule as applied to organ volume calculations

The two methods were compared for approximate equality of results as a quality control check. The subsequent statistical analysis was then performed on the interpolated volume measurements.

In the case of the seminal vesicle (SV) volume, the drawn ROIs contained fluid and organ wall in a convoluted pattern which was difficult to outline manually. To calculate a more accurate value for fluid volume, rather than use the whole ROI area in the organ volume calculation, a threshold was applied to yield a sub-set of areas to include within the drawn ROI. The threshold on image intensity was set a fraction (*f*) of the maximum pixel intensity (*S*_max_) calculated as the 95% percentile in the SV ROI pixel intensity histogram. The 95% percentile was used to avoid outlying values (noisy ‘hot-spots’) having an appreciable effect on the maximum signal recorded. Thus, for any given organ, the 95% maximum signal in the central outlined slice was found and the threshold for inclusion of pixels set to S > (*S*_max_ × *f*). The fraction *f* was varied manually between limits of 0.6 and 0.8 until optimal segmentation was achieved as evaluated through visual inspection by a radiologist. Fluid volume was then calculated in the same way as outlined above for complete organs, however in this case using the sum of thresholded ROI sub-areas as *y*-values in Simpson’s Rule together with the spacing between slices as ‘*h*’.
