# Supplementary material for: The longitudinal effect of ejaculation on seminal vesicle fluid volume and whole-prostate ADC as measured on prostate MRI
Source: Eur Radiol. 2017 Jul 4;27(12):5236–43. doi: 10.1007/s00330-017-4905-x (PMC5674119; doi:10.1007/s00330-017-4905-x)
Supplement: Supplementary file 3 — (DOCX 23 kb) [file 330_2017_4905_MOESM3_ESM.docx]

**Supplemental Table 1. Mean, range and standard deviation of whole gland ADC values for each volunteer and all studies**

| **Study** | **Mean ADC value** | **Minimum ADC value** | **Maximum ADC value** | **Standard deviation** |
| --- | --- | --- | --- | --- |
| Patient 1 – study 1 | 1.078 | 0.981 | 1.149 | 0.053 |
| Patient 1 – study 2 | 0.828 | 0.770 | 1.238 | 0.148 |
| Patient 1 – study 3 | 1.001 | 0.924 | 1.058 | 0.036 |
| Patient 1 – study 4 | 1.047 | 0.994 | 1.234 | 0.063 |
| Patient 2 – study 1 | 0.972 | 0.932 | 1.031 | 0.030 |
| Patient 2 – study 2 | 0.985 | 0.951 | 1.095 | 0.060 |
| Patient 2 – study 3 | 0.980 | 0.938 | 1.091 | 0.042 |
| Patient 2 – study 4 | 0.977 | 0.964 | 1.023 | 0.020 |
| Patient 3 – study 1 | 1.032 | 0.878 | 1.097 | 0.072 |
| Patient 3 – study 2 | 1.049 | 0.913 | 1.139 | 0.081 |
| Patient 3 – study 3 | 0.987 | 0.794 | 1.085 | 0.089 |
| Patient 3 – study 4 | 0.947 | 0.700 | 1.204 | 0.145 |
| Patient 4 – study 1 | 1.163 | 0.947 | 1.305 | 0.104 |
| Patient 4 – study 2 | 1.202 | 1.155 | 1.307 | 0.043 |
| Patient 4 – study 3 | 1.224 | 1.192 | 1.394 | 0.061 |
| Patient 4 – study 4 | 1.229 | 1.221 | 1.378 | 0.057 |
| Patient 5 – study 1 | 1.183 | 0.878 | 1.280 | 0.144 |
| Patient 5 – study 2 | 1.068 | 0.913 | 1.303 | 0.160 |
| Patient 5 – study 3 | 1.034 | 0.890 | 1.171 | 0.109 |
| Patient 5 – study 4 | 1.074 | 0.827 | 1.246 | 0.150 |
| Patient 6 – study 1 | 1.062 | 1.005 | 1.220 | 0.069 |
| Patient 6 – study 2 | 1.027 | 0.898 | 1.254 | 0.116 |
| Patient 6 – study 3 | 1.043 | 0.930 | 1.261 | 0.091 |
| Patient 6 – study 4 | 1.077 | 0.970 | 1.205 | 0.076 |
| Patient 7 – study 1 | 1.058 | 0.869 | 1.170 | 0.090 |
| Patient 7 – study 2 | 1.047 | 0.898 | 1.119 | 0.084 |
| Patient 7 – study 3 | 1.021 | 0.993 | 1.069 | 0.029 |
| Patient 7 – study 4 | 1.029 | 1.005 | 1.091 | 0.030 |
| Patient 8 – study 1 | 1.121 | 1.029 | 1.189 | 0.043 |
| Patient 8 – study 2 | 1.058 | 1.059 | 1.120 | 0.021 |
| Patient 8 – study 3 | 1.075 | 1.058 | 1.163 | 0.031 |
| Patient 8 – study 4 | 1.068 | 1.035 | 1.133 | 0.034 |
| Patient 9 – study 1 | 1.363 | 1.091 | 1.481 | 0.135 |
| Patient 9 – study 2 | 1.325 | 1.105 | 1.450 | 0.112 |
| Patient 9 – study 3 | 1.319 | 1.154 | 1.428 | 0.100 |
| Patient 9 – study 4 | 1.197 | 1.073 | 1.350 | 0.096 |
| Patient 10 – study 1 | 1.155 | 1.128 | 1.233 | 0.037 |
| Patient 10 – study 2 | 1.127 | 1.080 | 1.228 | 0.052 |
| Patient 10 – study 3 | 1.209 | 1.127 | 1.272 | 0.047 |
| Patient 10 – study 4 | 1.022 | 0.960 | 1.209 | 0.075 |
| Patient 11 – study 1 | 1.258 | 1.085 | 1.373 | 0.091 |
| Patient 11 – study 2 | 1.127 | 1.006 | 1.276 | 0.090 |
| Patient 11 – study 3 | 1.193 | 1.036 | 1.346 | 0.103 |
| Patient 11 – study 4 | 1.203 | 1.074 | 1.349 | 0.091 |
| Patient 12 – study 1 | 1.100 | 1.048 | 1.316 | 0.085 |
| Patient 12 – study 2 | 1.110 | 1.044 | 1.177 | 0.045 |
| Patient 12 – study 3 | 1.112 | 1.059 | 1.188 | 0.053 |
| Patient 12 – study 4 | 1.084 | 0.958 | 1.192 | 0.078 |
| Patient 13 – study 1 | 1.098 | 1.077 | 1.235 | 0.057 |
| Patient 13 – study 2 | 1.101 | 1.085 | 1.236 | 0.054 |
| Patient 13 – study 3 | 1.107 | 1.081 | 1.199 | 0.039 |
| Patient 13 – study 4 | 1.076 | 1.048 | 1.202 | 0.060 |
| Patient 14 – study 1 | 1.198 | 1.024 | 1.384 | 0.123 |
| Patient 14 – study 2 | 1.098 | 1.016 | 1.291 | 0.098 |
| Patient 14 – study 3 | 1.173 | 1.093 | 1.304 | 0.073 |
| Patient 14 – study 4 | 1.185 | 1.102 | 1.333 | 0.080 |
| Patient 15 – study 1 | 0.989 | 0.957 | 1.185 | 0.069 |
| Patient 15 – study 2 | 0.954 | 0.917 | 1.020 | 0.036 |
| Patient 15 – study 3 | 0.982 | 0.856 | 1.064 | 0.062 |
| Patient 15 – study 4 | 1.022 | 0.908 | 1.180 | 0.083 |
